# Dynamics of self-sustained activity in random networks with strong synapses

**DOI:** 10.1186/1471-2202-12-S1-P89

**Published:** 2011-07-18

**Authors:** Håkon Enger, Tom Tetzlaff, Birgit Kriener, Marc-Oliver Gewaltig, Gaute T Einevoll

**Affiliations:** 1 Dept. of Mathematical Sciences and Technology, Norwegian University of Life Sciences, NO-1432 Ås, Norway; 2 Honda Research Institute Europe GmbH, D-63073 Offenbach/Main, Germany; 3 Bernstein Center for Computational Neuroscience, D-79104 Freiburg, Germany

## 

**Figure 1:**
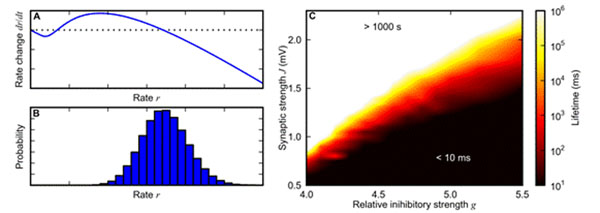
**A.** Phase space of the firing-rate dynamics. Dependence of the rate change *dr/dt* on the rate *r* (sketch). **B.** Firing-rate distribution in the self-sustained state (sketch). **C.** Dependence of the lifetime of self-sustained activity (color coded) on the level *g* of inhibition and the synaptic weight *J* in a random network of 100000 excitatory and 25000 inhibitory IaF neurons with 1% connectivity (simulation results).

An understanding of short-term memory requires models of neural networks which are able to sustain activity in the absence of external input for several seconds. About half a century ago, [1] predicted the existence of self-sustained activity in neural networks with strong synapses. Despite this finding, most previous studies on the dynamics of neural networks are restricted to weak synapses, i.e. to a regime where the diffusion approximation is applicable. Recently, it has been shown in simulations that self-sustained activity emerges in networks of integrate-and-fire (IaF) neurons with strong synapses modeled as currents [2] (rather than as conductances, see [3]). Above a critical synaptic weight, the lifetime of self-sustained activity increases rapidly (Fig. 1C). We present a stochastic model of the dynamics of a balanced random network of IaF neurons with current-based synapses in the strong-synapse regime. Based on the network's firing-rate transfer [4], we show that the firing-rate dynamics becomes bistable if the synapses are sufficiently strong: in addition to the quiescent state, a second stable fixed point at moderate firing rates is created (sketched in Fig. 1A). Firing-rate fluctuations can destabilize this fixed point, thereby limiting the lifetime of self-sustained activity (sketched in Fig. 1B). The magnitude of these fluctuations is mainly determined by the amount of spike-train correlations [5]. Our model explains the existence and the lifetime of self-sustained activity, and how the lifetime depends on the network size, the connectivity, the level of inhibition and the synapse strength. The results of our model are confirmed by network simulations.
